# Non-Invasive markers for hepatic fibrosis

**DOI:** 10.1186/1471-230X-11-91

**Published:** 2011-08-17

**Authors:** Ancha Baranova, Priyanka Lal, Aybike Birerdinc, Zobair M Younossi

**Affiliations:** 1 Betty and Guy Beatty Center for Integrated Research, Inova Health System, Falls Church, VA, USA; 2 Center for the Study of Genomics in Liver Diseases, School of Systems Biology, College of Science, George Mason University, Fairfax, VA, USA; 3 Center for Liver Diseases, Inova Fairfax Hospital, Falls Church, VA, USA

## Abstract

With great advancements in the therapeutic modalities used for the treatment of chronic liver diseases, the accurate assessment of liver fibrosis is a vital need for successful individualized management of disease activity in patients. The lack of accurate, reproducible and easily applied methods for fibrosis assessment has been the major limitation in both the clinical management and for research in liver diseases. However, the problem of the development of biomarkers capable of non-invasive staging of fibrosis in the liver is difficult due to the fact that the process of fibrogenesis is a component of the normal healing response to injury, invasion by pathogens, and many other etiologic factors. Current non-invasive methods range from serum biomarker assays to advanced imaging techniques such as transient elastography and magnetic resonance imaging (MRI). Among non-invasive methods that gain strongest clinical foothold are FibroScan elastometry and serum-based APRI and FibroTest. There are many other tests that are not yet widely validated, but are none the less, promising. The rate of adoption of non-invasive diagnostic tests for liver fibrosis differs from country to country, but remains limited. At the present time, use of non-invasive procedures could be recommended as pre-screening that may allow physicians to narrow down the patients' population before definitive testing of liver fibrosis by biopsy of the liver. This review provides a systematic overview of these techniques, as well as both direct and indirect biomarkers based approaches used to stage fibrosis and covers recent developments in this rapidly advancing area.

## Review

Liver fibrosis is defined as the building up of excessive amount of extracellular matrix, also known as scar tissue, in the liver parenchyma. While reviewing fibrosis as a component of the pathogenesis of a disease, it is important to remember that the process of fibrogenesis is also a component of the normal healing response to various kinds of injury. In the liver, this healing process normally involves the recruitment of immune and/or inflammatory cells to the site of injury, secretion of extracellular matrix (ECM) proteins, reorganization of the ECM and possible regeneration of the hepatic tissue. When the damage to the liver is chronic, excess fibrous connective tissue accumulates. In time, this process eventually distorts the normal parenchymal structure of the liver and impairs its function. As chronic liver disease progresses, hepatic fibrosis is accompanied by the formation of septae and nodules that intervene with the portal blood flow, leading to hypertension and formation of distinctive cirrhotic architecture. At all stages of the fibrogenesis, the stress exerted on the liver parenchyma is exemplified by subsequent activation of the immune system accompanied by increased levels of certain cytokines and growth factors, which augment fibrogenesis. In proinflammatory fibrotic microenvironment, constant stimulation of hepatocellular regeneration could predispose to the development of hepatocellular carcinoma (HCC). However disruptive, hepatic fibrosis even early cirrhosis can be reversed by suppression of the fibrotic response [1,2].

## The Biology of Liver Fibrosis

The most important cellular player in the production of the extracellular matrix is the myofibroblast (MF). A wide array of cells of different origins can be converted into fibrogenic MFs, including portal MFs and bone marrow-derived mesenchymal stem cells. Some epithelial cells including hepatocytes and biliary epithelial cells (cholangiocytes) could be activated to function as myofibroblasts through the process of Epithelial-Mesenchymal Transition (EMT) [3]. However, the predominant MF-producing liver cells are quiescent hepatic stellate cells (HSC), also known as Ito cells or perisinusoidal cells residing in the space of Disse and storing retinoids [4].

Non-MF cells actively participate in the process of fibrogenesis. For example, hepatocytes can respond to this damage in multiple ways, including production of reactive oxygen species (ROS) and apoptosis, while the resident liver macrophages called Kupffer cells elicit a massive immune response resulting in the recruitment of other inflammatory cells to the site of injury [5]. Attracted to the chemokines produced by the Kupffer cells, the leukocytes exit out of the vasculature towards the injury site and contribute to the release of additional pro-inflammatory and pro-fibrotic mediators, including cytokines such as tumor necrosis factor alpha (TNF-α) and various interleukins. Reactive oxygen and nitrogen species, proteases, and lipid metabolites such as prostaglandins and thromboxane are also released [6]. As a result of this response, quiescent HSCs are converted to activated myofibroblasts [7] and, in turn, contribute to the chemotaxis of leukocytes as well as their own chemotaxis through the production of chemokines and cytokines such as monocyte chemotactic protein-1 (MCP-1) [8]. As a result, activated HSCs start expressing the Platelet Derived Growth Factor (PDGF) receptor and Transforming Growth Factor (TGF) receptor. TGF-β is the central mediator of fibrogenesis, while PGDF stimulates proliferation of the HSCs. Activation of HSCs is associated with a gradual replacement of the basement membrane-like extracellular matrix (ECM) within the space of Disse by the collagen rich fibers [7] and the production of fibrous bands [8]. In advanced stages of fibrosis, the liver contains approximately six times more ECM components than normal, including collagens (I, III, and IV), fibronectin, undulin, elastin, laminin, hyaluronan, and proteoglycans [8].

As can be surmised from existing evidence, a number of functionally diverse biomolecules could be developed as biomarkers for hepatic fibrosis. One can look for biomarkers among molecules that change expression in the process of HSC activation or at the methods that enumerate MFs or their products. Another approach can look at direct quantification of ECM within the liver, or the particular molecules involved in the process of profibrogenic inflammation within the liver parenchyma. For any of these biomarkers, the clinical utility in the setting of the liver disease cannot be derived from the functionality of the respective molecules, thus, emphasizing the importance of extensive validation. Moreover, as the types of liver fibrosis differ in the fibrogenic mechanisms and in the distribution of the damage within the liver, each biomarker or panel thereof should be evaluated across a variety of clinical cohorts.

## Types and Causes of Acquired Liver Fibrosis

Acquired fibrosis may result from the action of a number of pathogenic factors and toxic exposures such as long-term excessive alcohol consumption, cholestasis, autoimmune liver diseases, iron or copper overload, chronic viral hepatitis or the presence of non-alcoholic fatty liver disease (NAFLD). These etiological factors may work separately or in combination with each other to produce cumulative effects. In this review, we will discuss the types of damage associated with the most common causes of acquired hepatic fibrosis and the biomarkers being developed in order to quantify and stage them. It is important to note that the presence of fibrotic changes and even significant fibrosis in different etiological contexts has different clinical meanings. For example, in the case of hepatitis C the presence of fibrotic changes could argue for the need for antiviral treatment, while in the case of NAFLD it does not change the therapeutic options, but may provide important prognostic information.

### Alcoholic Liver Disease

Excessive and chronic alcohol consumption is an important causal factor of liver fibrosis and cirrhosis. The process of the breakdown of ethanol produces two pro-fibrotic agents, acetaldehyde and reactive oxygen species (ROS). In hepatocytes, the primary site for alcohol metabolism, acetaldehyde and ROS are produced in abundance, then they diffuse outside and enter HSCs. Acetaldehyde directly up-regulates the transcription of collagen I [9] and the synthesis of transforming growth factor-beta 1 (TGF-β1). Exposure to ROS sensitizes HSCs to various pro-inflammatory factors and elicits the production of inflammatory mediators that contribute to the fibrotic changes in the liver [10]. In ALD, the fibrotic changes in the liver start in the pericentral and perisinusoidal areas [9,10].

### Non-alcoholic Fatty Liver Diseases (NAFLD)

NAFLD and its subtype, Non-Alcoholic Steatohepatitis, or NASH, are usually seen in individuals with metabolic syndrome (MS) or its components such as obesity, type-2 diabetes (DM), dyslipidemia, and insulin resistance. NASH rarely manifests as inflammation and/or apoptosis/necrosis only, more often than not it is also accompanied by liver fibrosis. To date, the pathogenesis of NASH-related liver fibrosis is not entirely well understood [8]. Evidence provided by numerous studies links obesity, insulin resistance and the progression of fibrosis together in one vicious circle [11]. The same factors are also known for their association with hepatic fibrosis. For example, leptin, the well-know adipokine produced proportionally to the mass of the visceral adipose compartment, also augments fibrogenesis by stimulating phagocytic activity and cytokine secretion by Kupffer cells and macrophages [12] as well as the proliferative and ROS generating activities of the endothelial cells [13]. Another adipokine, resistin, exerts pro-inflammatory actions in HSC by increasing the expression of both MCP-1 and interleukin-8 (IL-8) as well as activating the transcription factor, NFkB [14]. From examples mentioned above, one can derive that the initial stages of the pathogenesis of liver fibrosis associated with NAFLD depends primarily on the soluble factors produced by excessive visceral adipose and on a skewed distribution of the soluble fat particles in the bloodstream.

### Cholestatic Liver Diseases

Cholestasis (reduced bile duct excretion) is another well-known cause of liver fibrosis. Cholestasis triggers the proliferation of the cholangiocyte lining of the intrahepatic and extrahepatic bile duct systems through a complex regulatory milieu that involves both autocrine and paracrine factors [15]. The activation of biliary proliferation is known as ductular reaction. Proliferating bile duct epithelial cells produce the profibrogenic connective tissue growth factor (CTGF) that stimulates myofibroblast generation through EMT and collagen deposition [16]. The primary players in the fibrotic reaction to cholestasis are the inflammatory responses propagated by neutrophils and resulting from this oxidative stress. The fibrotic reaction is initiated in the portal area of the liver normally enriched in fibroblasts available for MF conversion [9,10]. The layer of hepatocytes adjacent to these fibroblasts is liable to immediate destruction leading to the enlargement of the portal field and rapid activation of the portal fibroblasts [10].

### Chronic Viral Hepatitis

Chronic viral infections such as hepatitis B (HBV) or hepatitis C (HCV) viruses pose an important risk for the development of liver fibrosis. The general mechanism of the fibrogenesis in chronic viral hepatitis is less clear than that in non-viral chronic diseases. In chronic viral hepatitis, fibrosis is usually initiated in the portal area [9]. Most likely, the pathogenesis is multifactorial as it involves a combination of both viral and host-specific factors, including oxidative stress, hepatic steatosis, increased iron stores, and increased rate of hepatocyte apoptosis, under the pressure of the viral proteins and viral replication.

In chronic HCV infection, the viral core, NS5 and NS3 proteins have been demonstrated to initiate a cascade of molecular events that can eventually lead to fibrosis. HCV proteins appear to affect both lipid accumulation and degradation, with the consequent disruption of the normal process of lipid compartmentalization and metabolism, skewing towards ROS production. In the case of HBV infection, studies have shown that the X protein of HBV (HBx) directly induces TGF-β secretion by hepatocytes and, thus, contributes to the paracrine activation of HSC's [17]. Both HIV-HBV and HIV-HCV co-infected patients are at increased risk for progression of their liver disease as compared to patients who are mono-infected with HCV or HBV [18].

## Diagnostics of Acquired Liver Fibrosis

With the advancements in the treatment of patients with chronic liver diseases, the accurate assessment of liver fibrosis has become increasingly important as it allows for individualized management. The lack of accurate, reproducible and easily applied methods for assessment of hepatic fibrosis has been the major limitation for both the clinical management and research in liver diseases.

**Figure 1:**
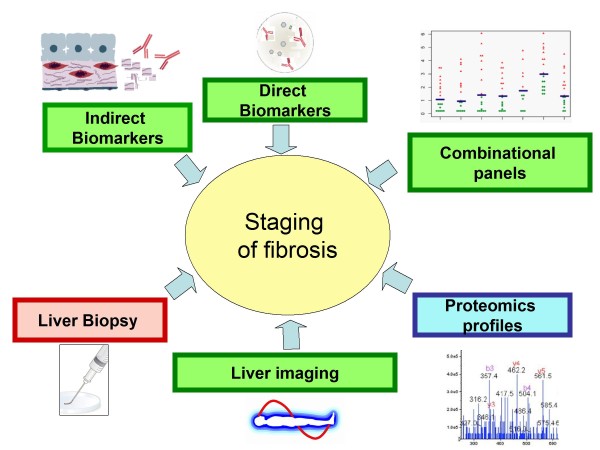
**A scheme depicting various means of liver fibrosis diagnostics**. Liver biopsy is an invasive method that remains an imperfect golden standard. Proteomics based profiles are unlikely to be introduced to routine clinical care anytime soon, but are valuable from the research point of view. Imaging techniques, serum biomarkers and biomarker panels are advancing along the route to the clinic stage, but require extensive validation.

The following paragraphs summarize the current modalities used for quantifying and staging hepatic fibrosis (Figure 1).

### Liver biopsy scoring techniques

For the past 50 years liver biopsy has been considered to be the gold standard for staging of liver fibrosis. This technique allows physicians to obtain diagnostic information not only on fibrosis, but also on many other liver injuring processes, such as inflammation, necrosis, steatosis, hepatic deposits of iron or copper. However, many recent studies clearly highlight several crucial drawbacks of liver biopsy, including variable accessibility, high cost, sampling errors and inaccuracy due to inter- and intra-observer variability of pathologic interpretations [19]. In addition, there is a small but important risk of liver biopsy-associated morbidity and mortality, with pain and hypotension as the most frequent complications and intraperitoneal bleeding and injury to the biliary system as the most serious complications. Studies reveal that the risk for hospitalization after liver biopsy is 1-5%, the risk for severe complications is 0.57%, and mortality rates vary from 0.009% to 0.12% [20,21]. Because of these reasons, some patients may opt to forgo liver biopsy and may not know the stage of their liver disease with important prognostic implications.

The history of the fibrosis scoring systems dates back to 1981 when the histological features of chronic hepatitis were evaluated for potential importance in determining its prognosis by Knodell and colleagues [22]. The Ishak score, or revised Knodell system, has primarily been applied to chronic hepatitis B and C. It considers grading and staging as two separate items; liver fibrosis is classified as: 0 = absent, 1-2 = mild, 3-4 = moderate and 5-6 = severe/cirrhosis. The first three axes of Knodell HAI (Histologic Activity Index) relate to the necroinflammatory grade of the disease while the fourth feature assesses the stage of the disease by evaluating the degree of fibrous portal tract expansion, fibrous portal-portal linking, portal-central fibrous bridges, and the formation of fibrous septa and parenchymal nodules [23]. This grading system has been subsequently modified by other pathologists [24,25]. There are some limitations of HAI index, in particular, related to the interobserver variation [26]. Another limitation of the Ishak/Knodell fibrosis score is its nonlinearity as it includes scores 0, 1, 3, and 4.

The Metavir scoring system was designed specifically for patients with hepatitis C using a sum of experience-based opinions of 10 pathologists augmented by subsequent stepwise discriminant analysis [27]. The scoring uses both grading and staging systems as it includes two separate scores, one for necroinflammatory grade (A for activity) and another for the stage of fibrosis (F). The grade is a number based on the degree of inflammation, which is usually scored from 0-4, with A0 being no activity and A3 to A4 considered severe activity. Determining the amount of inflammation is important because it can correlate with hepatic fibrosis. The degree of activity is assessed by the integration of the severity of both (periportal) necrosis and lobular necrosis as described in a simple algorithm [28]. The fibrosis score (F) is defined as: F0 = no scarring, F1 = portal fibrosis without septa, F2 = portal fibrosis with rare septa, F3 = numerous septa without cirrhosis and F4 = cirrhosis or advanced scarring of the liver [29]. The intra- and inter-observer variability of Metavir seems to be improved [30]. The main advantage of the Metavir score for hepatitis C is its relative simplicity, its focus on necroinflammatory lesions, and its increased sensitivity in the fibrosis score due to the addition of one extra fibrosis evaluating level.

However, the limitations of the Knodell score also apply to the Metavir score as it retains the semi quantitative and categorical nature of fibrosis staging. Use of the liver biopsy scoring systems often varies among different pathology laboratories, which makes score comparisons among patients from different centers rather difficult. Built-in sampling error problems associated with accepted scoring systems requires the need to design studies with extremely large sample sizes [31].

In addition to staging hepatic fibrosis for viral hepatitis, three pathologic criteria have been used for patients with NAFLD. Of these, the original classification of NAFLD subtypes was developed to histologically categorize NAFLD into 4 subtypes): type 1 NAFLD = steatosis alone; type 2 NAFLD = steatosis with lobular inflammation only; type 3 NAFLD = steatosis with hepatocellular ballooning; or type 4 NAFLD = steatosis with Malloy-Denk bodies or fibrosis. According to these criteria, types 3 and 4 NAFLD were considered to be NASH. Subsequently, Brunt's criteria was developed to grade NASH and used for clinical research in patients with NAFLD. According to Brunt's criteria, liver biopsy with at least fat and lobular inflammation is graded as mild (grade 1), moderate (grade 2) or marked (grade 3) NASH. More recently, the NAFLD Activity Score (NAS) was developed to provide a numerical score for patients who most likely have NASH. Accordingly, NAS is the sum of the separate scores for steatosis (0-3), hepatocellular ballooning (0-2), lobular inflammation (0-3), with most patients with NASH having a NAS score of ≥5. Fibrosis, according to both Brunt and NAS, is scored from 0 to 4 (grade 0 = none; 1 = centrilobular/perisinusoidal; 2 = centrilobular plus periportal; 3 = bridging; 4 = cirrhosis) [32]. These pathologic protocols for NAFLD suffer from a lack of data assessing their inter-observer variability as well as their inability to predict liver-related mortality.

To overcome the previously mentioned complications posed by liver biopsy, alternative non-invasive methods for quantifying and staging liver fibrosis have been developed. These methods range from serum biomarker assays to advanced imaging techniques (Figure 1 and Table 1).

**Table 1:** Serum biomarkers and imaging techniques for the detection of liver fibrosis: A summary of the features

Feature	Serum biomarkers	Imaging techniques
Invasiveness	Minimal (venopuction)	None

Sensitivity to sampling error	Minimal	Some

Interpretability of the test	High, if instructions are closely followed	Depends on experience of operator

Costs per test	Depend on a type of the test, but generally comparable with tests for serum insulin	Depends on the cost of equipment: highest for MRI. lowest for ARFI

Limitations imposed by anthropometric features	None	For TE and ARFI: width of the inter-costal space, the presence of ascites, the body mass index of patient and presence of visceral adiposity For MRI: fitting within the magnetic bore, severe hemochromatosis, claustrophobia

Possibility of multiplexing	Possible, but often hard to implement	Not applicable

Suitability for longitudinal monitoring	High	High

Accuracy for the prediction of cirrhosis	Moderate-to-High	High

Accuracy for the prediction of adjacent stages of fibrosis	Low-to-Moderate	Low-to-Moderate

Interference by necroinflammatory activity and steatosis	Variable depending on the type of the test	Detectable for TE and ARFI

It is important to note that the performance of each non-invasive method, serum- or instrument based, is evaluated against a properly scored liver biopsy that continues to serve as an imperfect but indispensable standard for comparative studies of liver fibrosis diagnostics. Every non-invasive test is evaluated using the area under the ROC curve (AUC) that combines the sensitivity and specificity of a given quantitative marker for the diagnosis of a specific definition of fibrosis. For each test, sensitivity and specificity are usually assessed by enumerating correct diagnoses of advanced fibrosis (i.e., stages F2, F3, and F4 in the METAVIR scoring system) versus minimal or non-advanced fibrosis (i.e., stages F0, F1). Recently, the prevalence of each of the fibrosis stages, considered advanced or non-advanced fibrosis, in study cohorts was found to be highly associated with the AUC estimates [33]. Strikingly, AUCs for a typical study were shown to fluctuate in a range from 0.67 to 0.98 for the same test and the same type of liver disease depending on the distribution of stages within the cohort [33]. This means that AUCs obtained in different studies should not be compared directly, but a unifying correction for the stage distribution should be performed first.

Obviously, this fact reflects on the conclusions that can be made by comparison of the AUC-evaluated performances of non-invasive tests. Moreover, it is important to bear in mind that the biopsy itself has an AUC, which could be derived from the "true gold standard" studies of entire section of the liver [34]. Due to liver biopsy sampling variability, the discrimination of the adjacent fibrosis stages by METAVIR scoring on typical 15-mm core is correct in approximately 65% of cases, reflecting in AUC of only 0.82 when compared with the entire liver [34]. Therefore, the performances of non-invasive biomarkers with AUCs comparable to liver biopsy' own AUC are difficult to compare and verify. In the absence of studies involving whole liver sectioning, some of the proposed candidate biomarker may, in fact, reflect the overall fibrosis within the liver better than the liver biopsy itself.

**Figure 2:**
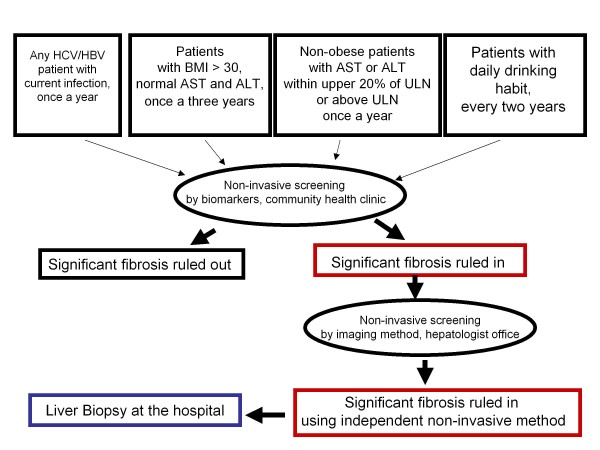
**Decision tree that may help to guide the cost-effective diagnosis of liver fibrosis in large populations of prospective patients**.

The rate of adoption of non-invasive diagnostics for liver fibrosis differs from country to country, but remains limited. At present, the use of non-invasive procedures could be recommended as pre-screening tools, which may allow physicians to narrow down the patients' population before definitive testing of liver fibrosis by biopsy of the liver (Figure 2).

### Imaging Techniques

The activation of HSCs and deposition of the ECM leads to alterations in liver microstructure that are reflected by an increase in the liver stiffness and changes in the blood flow. Recent radiological advances allow the bedside assessment of liver stiffness with techniques like Fibroscan, ARFI and MRI.

#### Transient Ultrasound Elastography (FibroScan)

An ultrasound-based technology for quantitatively assessing hepatic stiffness has been introduced in the last several years both in Europe and other parts of the world and is consistently gaining traction. FibroScan measures the stiffness (or elasticity) of the hepatic parenchyma using both ultrasound (5 MHz) and low-frequency (50 Hz) elastic waves produced by a specialized ultrasound vibrator applied to the body wall and coupled with 1D ultrasound imaging that measures the propagation speed of a wave using a pulse-echo ultrasound. Since fibrotic tissue is harder than healthy liver tissue, the shear wave measurement provides immediate quantitative assessment of the "degree of stiffness" which takes less than 5 minutes to collect. Most of the FibroScan studies originate in Europe where this method was invented and approved for use in routine clinical settings. FibroScan was reported to be of value in the diagnosis of the fibrosis accompanying various liver diseases including hepatitis B and C, alcoholic liver disease, and non alcoholic fatty liver disease (NAFLD) [35,36]. By meta-analysis, FibroScan is considered to be a reliable method for the diagnosis of significant fibrosis (AUC = 0.84), severe fibrosis (0.89), and cirrhosis (0.94) [36]. However, for the diagnosis of significant fibrosis, there was a high variation of the AUCs dependent on the underlying liver disease [36]. In a recent multicenter prospective study, FIBROSTIC, FibroScan measurements predicted cirrhosis with higher AUROCs (0.89 - 0.90) than serum based biomarkers (AUROC 0.77-0.86) [37].

Similar to serum-based tests, transient elastography can be repeated over time, thus, providing an opportunity for longitudinal disease monitoring and comparative assessments that are difficult when the primary diagnostic means is a liver biopsy. FibroScan technology is not free of limitations. Most importantly, its accuracy in predicting significant cirrhosis is influenced by necroinflammatory activity and steatosis common in patients with NAFLD [38]. To improve the performance of FibroScan, its combination with serum based FibroMeter test was suggested [39]. According to large series of liver stiffness measurements that included 13,369 individual examinations, these measures remain impossible to interpret in nearly one in five cases [40]. The success rate of the procedure is dependent on observer expertise as well as on age of the patient, the width of the inter-costal space, the presence of ascites, the body mass index of patient and presence of visceral adiposity [40,41]. Accordingly, the diagnostic performance of transient ultrasound elastography is much lower in patients with early-stage hepatic fibrosis, increased fatty infiltration of the liver on biopsy, or high body mass index (≥28 kg/m^2^) [42].

#### Acoustic radiation force impulse (ARFI)

ARFI imaging combines conventional unltrasonography of the liver with evaluation of local liver stiffness. As regions of evaluation can be chosen using ultrasound, ARFI allows operator avoidance of anatomical obstacles, e.g. large blood vessels [43,44]. In a small study of 99 patients with liver disease and 23 healthy controls, ARFI measurements were highly correlated to that of FibsroScan, but the rate of invalid measurements with ARFI was lower (P < 0.04). Importantly, in contrast to FibroScan, liver steatosis had no statistical influence on ARFI results [45]. Other studies compared ARFI to Forns' index and other non-invasive, clinical parameters-based diagnostic tests, and found that in hepatitis C patients ARFI' diagnostic ability was superior to any of these [46]. An important advantage of ARFI is that it can be performed with software integrated into conventional ultrasound equipment as opposed to purchasing expensive TE-enabling Fibroscan units.

#### Magnetic Resonance Imaging (MRI)

MRI technology can be used to measure both liver stiffness and characteristic water-diffusion abnormalities associated with cirrhosis [47]. It should not be confused with magnetic resonance spectroscopy (MRS), which provides images of the metabolic abnormalities in subjects with liver disease.

Several types of enhanced MRI have been developed to evaluate the degree of liver fibrosis. One such modification, Magnetic Resonance Elastography (MRE) directly visualizes and quantitatively measures acoustic shear waves progressing through the liver tissue. MRE involves a three-step process:1) generating mechanical waves within the tissues of interest; 2) imaging the micron level displacements caused by propagating waves using a special MRI technique with oscillating motion-sensitizing gradients; and 3) processing the wave images using an inversion algorithm to generate quantitative maps of the physical properties of the liver [48]. Importantly, MRI accurately reflects the distribution of the fibrous material in the liver, thus, at least in some cases helping with identification of the nature of injury [8].

The current evidence support the observation that a normal mean liver stiffness value by MRE in the setting of chronic liver disease is consistent with stage 0 fibrosis on liver biopsy [48], while fibrosis of stages 1-4 are also diagnosed accurately [48-50]. In a study encompassing 50 patients with biopsy-proven liver disease and 35 healthy volunteers, receiver operating characteristic (ROC) analysis showed that, with a shear stiffness cutoff value of 2.93 kPa, the predicted sensitivity and specificity for detecting liver fibrosis were 98% and 99%; respectively [49]. Notably, stage I fibrosis detection was possible; while the detection of hepatic fibrosis with stages 2 or more, the area under the ROC curve (AUC) was 0.96 [50]. It was also noted that the technical success rate for MRE is significantly higher than that of transient elastography (94% vs. 84%). The only practical limitation of performing MR elastography in very obese patients is that the patient has to fit within the magnetic bore, the diameter of which is also further reduced by the presence of the transducer.

It is important to note that MRE is associated with substantially higher costs than FibroScan or ARFI. Furthermore, MRE use can be limited for claustrophobic patients or those with severe hemochromatosis. These limitations preclude MRE from wide spread clinical acceptance and its primary purpose remains a research.

### Serum Biomarkers of Fibrosis

In recent years, interest in identifying and describing liver fibrosis by using non invasive surrogate markers has been on the rise. Serum markers of liver fibrosis offer an attractive, cost effective alternative to liver biopsy for both patients and clinicians. In addition to being substantially less invasive, there are practically no complications, little or no sampling errors and small observer related variability. Moreover, measurements may be performed repeatedly, thus, allowing for a dynamic monitoring of fibrosis [51].

A majority of commonly used biomarkers were identified in the past two decades in relatively low-throughput clinical studies centered on the pathogenic mechanisms. Recently, the process of biomarker discovery was augmented by a number of commercial high-throughput pipelines aimed at singling out the molecules differentially expressed between various physiological and pathological states followed by validation of their performance in independent cohorts and true translation from bench to bedside as pre-packaged diagnostic kits.

While the first step on this path, the discovery of new biomarker leads, produced a variety of promising candidates, assessing each biomarker in a statistically significant number of samples and controls still constitutes a major technical challenge. Another problem is the fair assessment of relative value of novel biomarkers or biomarker panels as compared to existing non-invasive predictors. Typically, the performance of each novel biomarker is compared to that of either one or more other panels, and the assessment is limited to one pathological condition (i.e. alcoholic liver disease). Given this approach, the exact contribution of each newly described biomarker to the prediction of liver fibrosis is difficult to evaluate. A recent study of Park and coauthors specifically addressed this question and concluded that the simultaneous addition of several biomarkers adds only modestly to clinical predictive factors for the risk assessment of individual patients [52].

#### The Ideal Biomarker of Liver Fibrosis

The diagnostic value of serum markers of liver fibrosis has been investigated in numerous studies. Based on clinical and research needs, the ideal marker for liver fibrosis would have the following characteristics:

• Be highly sensitive and specific to identify different stages of fibrosis

• Be readily available, safe, inexpensive and reproducible

• Be applicable to the monitoring of disease progression or regression as apart of natural history of liver disease or treatment regimens

• Not be susceptible to false positive results, for example, in individuals with inflammation related to other diseases

Although no single ideal marker exists, several markers have been identified as possible useful indicators of fibrosis when used in conjunction with each other.

Biomarkers of fibrosis are commonly divided into Direct and Indirect markers. Direct markers are fragments of the liver matrix components produced by hepatic stellate cells (HSC) during the process of ECM remodeling. Indirect markers include molecules released into the blood due to liver inflammation, molecules synthesized/regulated or excreted by the liver, and markers of processes commonly disrupted due to liver function impairment, such as insulin resistance (Table 2 and [53]). Direct and indirect markers may be used alone or - more commonly - in combination with each other, to produce composite scores. The calculation of such scores can be relatively simple or can be based on complicated formulas (e.g. those underlying Fibrotest/Fibrosure).

**Table 2:** Indirect Serum Markers of Liver Fibrosis.

Indices	Individual components	Sensitivity (%)	Specificity (%)
AST/ALT ratio	Aspartate aminotransferase, Alanine aminotransferase	53	100

PGA	Protrombin index, GGT, apolipoprotein A1	91	81

APRI	AST/platelet count	89	75

FibroSpect II	HA, TIMP-1, α2-macroglobulin	83.5	66.7

FibroTest/FibroSure	γ2 macroglobulin, γ2 globulin, γ globulin, apolipoprotein A1, GGT, total bilirubin	75	85

FibroIndex	Platelet count, AST, GGT	78	74

FibroMeter	Platelet count, γ2 macroglobulin, AST, age, prothrombin index, HA, blood urea nitrogen	81	84

Forns	Age, platelet count, GGT, cholestered levels	94	51

Hepascore	Age, gender, bilirubin, GGT, HA, γ2-macroglobulin	63	89

FIB-4	Platelet count, ALT, AST, platelet count, age	70	74

SHASTA Index	HA, AST, albumin	100	52

Simple test	age, hyperglycemia, BMI, platelet count, albumin, AST/ALT	78	58

OELF/ELF	age, HA, N-terminal propeptide of type III collagen, TIMP-1	90	41

GGT: γ glutamyl transferase, HA: hyaluronic acid, TIMP-1: Tissue inhibitors of matrix metalloproteinase- 1, ALT: Alanine aminotransferase, AST: Aspartate aminotransferase

The most commonly used markers are discussed in details below.

#### Direct Biomarkers

##### Procollagen type I carboxy terminal peptide (PICP) and Procollagen type III amino-terminal peptide (PIIINP)

In the healthy human liver the most abundant collagens are the fibril-forming types I and III. In its mature form, the collagen is integrated into the ECM. During fibrogenesis, type I collagen levels increase up to eightfold. Additionally, the ratio of the type I/III also changes from 1:1 in the healthy liver to 1:2 in the cirrhotic liver [54].

PIIINP is another major constituent of the connective tissue. Its relative concentration in the basement membrane is higher in hepatic fibrogenesis and is closely followed by an increase in its serum level [55]. In acute hepatitis, levels of serum PIIINP correlate with aminotransferase levels. In chronic liver disease, serum PIIINP reflects the stage of liver fibrosis [56]. Unfortunately, PIIINP is not specific for the fibrosis of the liver as it is also elevated in acromegaly, lung fibrosis, chronic pancreatitis, and rheumatologic disease [54].

PICP levels are normal in patients with mild chronic hepatitis C and elevated in 50% of patients with moderately advanced or advanced chronic hepatitis C, including patients with liver cirrhosis of this etiology [57]. However, there is no correlation between the levels of PICP and PIIINP.

##### Metalloproteinases (MMPs)

MMPs form a family of structurally related proteolytic enzymes that mediate the degradation of the ECM and the basal membranes [56,58]. The three most commonly studied human metalloproteinases are MMP-2 (gelatinase-A), MMP-3 (stromelysin), and MMP-9 (gelatinase-B). MMP-2 is secreted by activated HSCs; elevated levels of MMP-2 and its proenzyme have been observed in various liver diseases [59]. During hepatic fibrogenesis, the expression of MMP-2 is markedly increased. The potential for MMP-2 for predicting liver fibrosis remains unclear as some contradictory data have been reported by studies performed so far [60,61]. In contrast to MMP-2, MMP-9 levels show their value primarily in the diagnosis of hepatocellular carcinoma [62]. In one of the studies, MMP-9 levels were negatively correlated to the histological severity of the liver disease in patients with chronic hepatitis C [63].

##### Tissue inhibitors of matrix metalloproteinases (TIMPs)

TIMPs are secreted proteins that interact with MMPs and modulate their activation and functioning. TIMP-1 controls activity of most MMPs, whereas TIMP-2 specifically inhibits MMP-2. TIMPs-dependent inhibition of ECM degradation may promote liver fibrosis; elevation of TIMPs' levels has been observed in chronic liver disease. For example, chronic hepatitis C causes the elevation of both TIMP-1 and TIMP-2 in corollary with fibrosis progression [60]. A recent study of the relationship between serum MMP-9, TIMP-1 and fibrosis in 50 patients with various chronic liver disease showed that serum levels of MMP-9 in chronic hepatitis patients were low as compared to the controls (*P *< 0.05) [63]. Moreover, serum MMP-9 levels decrease as chronic hepatitis progresses to cirrhosis, while TIMP-1 levels increase along with an increase of the degree of fibrosis (r = 0.73, *P *< 0.001). These findings prompt using serum TIMP-1 as a non-invasive assay in liver fibrosis [63].

*Transforming growth factor-β1 (TGF-β1) *is a pleiotropic cytokine involved in tissue growth, differentiation, ECM production and the immune response. Three isoforms (β1, β2 and β3) of this cytokine have been identified, but only TGF-β1 is linked to liver fibrogenesis. TGF- β1 is also commonly accepted as a central component of fibrogenic response to wounding and is up-regulated in a variety of different diseases [8,54]. A correlation between TGF- β1 levels and the rate of fibrosis progression is widely accepted [41,64].

*Hyaluronic acid (HA) *is a glycosaminoglycan component of the ECM that is synthesized by the HSC. In a study of NAFLD-related fibrosis of the liver, HA was found to be the best class I biomarker of fibrosis, being associated with an area under curve (AUC) of 0.97 [65]. Since the negative predictive value of HA is much higher (98-100%) than its positive predictive value (61%), its main utility in its ability to rule out advanced fibrosis and cirrhosis [54].

*YKL-40 (chondrex) *is a mammalian homologue of the bacterial chitinases involved in remodeling or degradation of the extracellular matrix [66]. In liver diseases, serum levels of YKL-40 are closely related to the degree of histologically documented fibrosis [67].

*Laminin *is a major non-collagenous glycoprotein synthesized by the HSC and deposited in the basement membrane of the liver. During fibrosis, laminin accumulates around the vessels, in the perisinusoidal spaces and near the portal tract [68]. Elevated levels of laminin and pepsin resistant laminin (laminin P1) were found to correlate with the degree of perisinusoidal fibrosis [69].

*Connective tissue growth factor (CTGF) *is synthesized in response to profibrogenic factor TGF-β by both activated HSC and hepatocytes [70]. However, serum CTGF levels decrease in the end-stage cirrhosis [70].

*Paraoxonase 1 (PON-1) *is an enzyme that hydrolyzes lipid peroxides, has antioxidant properties and influences hepatic cell apoptosis. Measurement of serum PON1 activity has been proposed as a potential test for the evaluation of liver function, however, it clinical acceptance is limited due to instability and toxicity of its substrate, paraoxon [71]. Ferre ant coauthors [72] showed that baseline and stimulated PON1 activities are decreased in chronic hepatitis and in liver cirrhosis. The combination of baseline serum PON1 with five standard biochemical tests had higher classification accuracy (94% of patients; 96% of controls) than the five standard tests alone (75% of patients; 96% of controls). ROC analysis showed that AUROC for chronic hepatitis was 0.89 and was 0.96 for cirrhosis, both compared to controls [72].

*Microfibril-associated glycoprotein 4 *(*MFAP-4) *is a ligand for integrins. In a recent study, quantitative analysis of MFAP-4 serum levels showed high diagnostic accuracy for the prediction of non diseased liver versus cirrhosis (AUROC = 0.97, *P *< 0.0001) as well as stage 0 versus stage 4 fibrosis (AUROC = 0.84, *P *< 0.0001), and stages 0 to 3 versus stage 4 fibrosis (AUROC = 0.76, *P *< 0.0001) [73]

#### Limitations of Direct serum biomarkers of fibrosis

• They reflect the rate of matrix turnover (not only deposition) and have a tendency to be more elevated when associated with high inflammatory activity. As a consequence, extensive matrix deposition might not be detected in the presence of minimal inflammation;

• They are not liver-specific and their serum levels may be elevated in the presence of concomitant sites of inflammation;

• Serum levels of markers depend on their clearance rates, which are influenced by the dysfunction of endothelial cells, impaired biliary excretion or renal function [53].

### Indirect Biomarkers of Fibrosis

Most Indirect biomarkers of fibrosis are integrated with one or more fibrosis predicting biomarker panels.

#### AST/ALT ratio

Aspartate aminotransferase (AST) and Alanine aminotransferase (ALT) are hepatic enzymes that are released into the bloodstream from damaged hepatocytes. The predictive value of the AST/ALT ratio has been validated in non-alcoholic liver disease, chronic viral hepatitis, primary sclerosing cholangitis, and primary biliary cirrhosis [74]. In many forms of acute and chronic liver injury or steatosis (fatty infiltration of the liver), this ratio is less than or equal to 1, while in alcoholic hepatitis, an AST/ALT ratio is often greater than 2. While these ratios are suggestive of certain etiology of liver conditions, there is too much overlap between groups to rely on AST/ALT exclusively when making a diagnosis - for example, in patients with both hepatitis C and history of alcohol abuse [75].

The *PGA index *combines the measurement of the Prothrombin Index, γ glutamyl transferase levels and apolipoprotein A1. It was subsequently modified to the PGAA index by the addition of α2- macroglobulin, which resulted in marginal if any improvement in its performance. In chronic liver diseases, the PGA index has a relationship to both the inflammation and the fibrosis (*P <*0.01, *P <*0.05 respectively). However, overall accuracy of this index is relatively low [76,77].

The *AST-to-Platelet Ratio Index (APRI) *is calculated as (AST/upper limit of normal range)/platelet count (10^9^/L) × 100. This index has previously been validated as a surrogate marker of significant hepatic fibrosis in HIV/HCV-coinfected patients, and has recently been used to determine advanced fibrosis in HIV-monoinfected patients [78]. However, recent large meta-analysis suggested that APRI can identify hepatitis C-related fibrosis with only a moderate degree of accuracy [79].

The *Forns index *is based on 4 routine clinical variables: age, platelet count, cholesterol levels, and γ glutamyl transferase. This method can be used to differentiate patients with mild (F0-F1) fibrosis from those with severe (F2-F4) fibrosis, but it is less accurate in distinguishing patients with grades F2 versus F4. The Forns index has been validated in other cohorts as a predictive tool for response to anti-HCV therapy [80].

The *HepaScore *combines age, gender, bilirubin, γ glutamyl transferase, hyaluronic acid, and γ2-macroglobulin into a score from 0.00 to 1.00 [81]. In 512 chronic HCV patients, automated HepaScores showed good predictive performances for significant fibrosis (AUROC = 0.81), severe fibrosis (AUROC = 0.82), and cirrhosis (AUROC = 0.88). Importantly, HepaScore test can be automated using a single analyzer [81].

The *FIB-4 *score which combines platelet count, ALT, AST and age, was originally developed for use in HIV-HCV co-infection. Use of this index correctly classified 87% of patients with FIB-4 values outside 1.45-3.25 and avoided biopsy in 71% of the validation set with an AUROC of 0.765, sensitivity of 70% and a specificity of 97% for differentiating Ishak 0-3 from 4-6 [82]. This model was subsequently validated by Vallet-Pichard in a large cohort of HCV mono-infected patients, with the finding that using these ranges, 78% of 847 biopsies were correctly classified [AUROC 0.85 for severe fibrosis and 0.91 for cirrhosis] [83].

The *SHASTA Index *which consists of serum hyaluronic acid (HA), AST, and albumin was evaluated in a cohort of 95 patients with HIV/HCV co-infection [84]. Using a cut off of 0.8 resulted in a specificity of 100% and a positive predictive value of 100%, but this applied to less than 5% of patients. At the other end of the spectrum, a cutoff of less than 0.30 was associated with a sensitivity of more than 88% and a negative predictive value of more than 94%. Overall 42% of patients could be correctly classified at either extreme; however 58% were classifiable with scores between 0.3 and 0.8. However, the SHASTA index in HIV/HCV in this cohort has performed significantly better than the APRI test [85].

The *^13^C-methacetin breath test (MBT) *is amongst several ^13^C breath tests used for the quantitative non-invasive assessment of cytochrome P450-dependent hepatocellular function [86]. MBT is rapidly metabolized by healthy liver cells into acetaminophen and ^13^CO_2 _in a single dealkylation event, so the increase of ^13^CO_2 _in breath samples can be quantified by isotope ratio mass spectrometry or non dispersive isotope-selective infrared spectroscopy [86]. MBT has been shown to have high sensitivity (92.6%) and specificity (84.1%) in predicting liver cirrhosis. The areas under the curve were found to be 0.958 for predicting cirrhosis and 0.827 for identifying patients with advanced fibrosis [87]. MBT does not involve a blood test and can provide an immediate result at the point-of-care.

The *FIBROSpect II *test uses a combination of components in the fibrogenic cascade, such as hyaluronic acid, TIMP-1 (tissue inhibitor of metalloproteinase), and α-2-macroglobulin to calculate a composite score. The test is intended to differentiate mild fibrosis (Metavir stages F0 to F1) from more severe disease (Metavir stages F2 to F4), and had been shown to do well in chronic hepatitis C cohorts [88,89].

The *FibroTest *and *FibroSure *are identical tests marketed under different names in Europe and America for the assessment of fibrosis and necroinflammatory activity. The FibroTest score is computed by accessing a proprietary website and entering the patient's age, sex, and results for serum haptoglobin, α_2_-macroglobulin, apolipoprotein A1, γ-glutamyltransferase, and bilirubin analyses [90]. It generates a score that is correlated with the degree of liver damage in people with a variety of liver diseases. Due to the variability of component of assays and analyzers, FibroTest assays can only be performed in validated laboratories [91]. A recent study showed an AUROC of 0.69 and 0.91 for the diagnosis of significant fibrosis (F≥2) and liver cirrhosis in 74 patients comprising of 36 with HCV, 10 with HBV, and 28 with primary biliary cirrhosis [92]. The sensitivity and specificity values for FibroTest based detection of primary severe fibrosis were found to be 75% and 85%, respectively [77].

The *FibroIndex *was developed by Koda and co-authors [92] for liver fibrosis in chronic hepatitis C. This test relies on platelet count, AST and serum IgG. FibroIndex showed high predictive values for significant fibrosis, including in a subgroup of HCV cases with normal alanine aminotrasferase (NALT) [93]. The sensitivity and specificity of FibroIndex for detecting fibrosis in patients with HCV were 78% and 74% [94]. In a comparative study, the validated AUROC of the FibroIndex for predicting significant fibrosis was found to be 0.83 and 0.82, which is better than those of the Forns index and APRI in patients with chronic hepatitis C [94].

The *FibroMeter *is a combination of the platelet count, prothrombin index, AST, γ2 macroglobulin, hyaluronate, blood urea nitrogen and age. The good performance and applicability of FibroMeter was validated in a number of chronic liver diseases, including chronic viral hepatitis B or C, alcoholic liver disease (ALD) and non-alcoholic fatty liver disease (NAFLD). An important feature of the FibroMeter is that it presents the amount of liver fibrosis as a percentage of fibrous tissue within the liver. Another significant feature of FibroMeter is that it validates the results through an expert system that detects erroneous results. FibroMeter has two main diagnostic targets - fibrosis stage corresponding to the histological staging system Metavir and the amount of fibrosis which corresponds to morphometric determinations of the fibrotic area [95].

Two additional panels have been developed to assess hepatic fibrosis specifically in NAFLD. First, the so called "Simple Test" for fibrosis in NAFLD is a relatively easy to use panel that includes age, hyperglycemia, body mass index, platelet count, albumin, and AST/ALT [96]. When the purpose of performing a liver biopsy in NAFLD is to determine the extent of hepatic fibrosis, using the Simple Test can correctly stage 90% of patients, obviating the need for liver biopsy in approximately 75% of patients. In addition to the "Simple Test", another panel for hepatic fibrosis is the Original European Liver Fibrosis (OELF) panel [97], which includes age, hyaluronic acid, amino-terminal propeptide of type III collagen, and tissue inhibitor of matrix metalloproteinase 1. A simplified version OELF is ELF, which does not include age, and seems to perform well in patients with NAFLD [98]. The performances of ELF and OELF were found to be almost identical. In a recent study, when the ELF panel was used for "ruling in" severe fibrosis, only 14% of NAFLD patients in this cohort required a liver biopsy. The combination of Simple/ELF panel reached an AUROC of 0.98 for distinguishing severe fibrosis from initial stages of the fibrotic disease in patients with NAFLD [98].

The *Proteomics based tests *assess patterns of protein or glycoprotein by mass spectroscopy using serum samples. Importantly, while a series of 'peaks' generated, the precise identities of these peaks remain unknown. For example, Callewaert N et al., 2004 developed tests based on the altered N-glycosylation of total serum protein (GlycoCirrhoTest and GlycoFibroTest) [99], which could be both cost-effective and could rapidly determine a signature profile for n-glycans. At first, it was reported that the combination of GlycoCirrhoTest with the FibroTest produced a sensitivity of 79% and specificity of 86% in distinguishing cirrhosis from non cirrhotic disease. However, later tests showed limited applicability of the test to discern the etiology of liver diseases. Specifically, under galactosylation did not show a significantly different quantitative alteration in the cirrhotic and noncirrhotic population of all etiologies [100]. Moreover, the same modifications seem to continuously reappear in all liver diseases: hyperfucosylation, increased branching and a bisecting N-acetylglucosamine [100]. Larger prospective studies are necessary to determine the clinical application of these new technologies.

The recently developed *Phosphoproteomics *tests serve the goal of improving and understanding the pathogenesis of liver fibrosis to more than actually contributing to the practicality of clinical diagnostics. For example, phosphoproteomics based tests predict the fibrosis of the liver have been used to profile the phosphorylated (i.e. activated) forms of the major signaling proteins in visceral adipose samples of patients with NAFLD [101].

Recently, a number of attempts were made to improve sensitivity and specificity of non-invasive biomarker-based tests through combining them using sequential algorithms. One example of such studies is a work of Sebastiani et al, who combined APRI, Forns' index and Fibrotest to reduce need for liver biopsy in hepatitis C patients by 50-70% [102]. Later, the same group of authors combined APRI with FibroTest-FibroSure and validated this approach known as SAFE (sequential algorithm for fibrosis evaluation) biopsy in a very large cohort of hepatitis C patients (N = 2035) [103]. For the detection of fibrosis, SAFE biopsy had remarkable accuracy of 92.5% (95% confidence interval, 0.89-0.94), obviating the need for 81.5% of liver biopsies [103].

## Conclusions

Successful individualized management of chronic liver disease depends on the correct staging of liver fibrosis. In order to provide the means of monitoring the course of liver disease and its response to therapy, staging should be performed in a non-invasive and reproducible manner. The fact that the process of fibrogenesis is a component of the normal healing response hampers the development of disease-specific biomarkers; this is why the quest for suitable non-invasive biomarker of liver fibrosis has become one of the biggest challenges in translational hepatology. A number of non-invasive techniques ranging from serum biomarker assays to advanced imaging techniques are being developed. Among non-invasive methods that have gained the strongest clinical foothold are FibroScan elastometry and serum-based APRI and FibroTest. There are many other tests that are not yet widely validated, but remain promising. Major validation efforts to enroll large cohorts of patients with chronic liver diseases controlled for important confounders such as ethnicity, body mass index and etiology of liver disease, must be undertaken.

Importantly, most non-invasive tests fail to differentiate between early stages of fibrosis. In fact, most of these tests can primarily distinguish cirrhosis from no or minimal fibrosis. For the diagnosis of significant liver fibrosis (F > or = 2) non invasive tests cannot yet replace liver biopsy. Therefore, the current utility of non-invasive diagnostics remains limited to pre-screening allowing physician to narrow the population of patient before definitive testing of liver fibrosis by biopsy of the liver.

## Abbreviations

MRI: magnetic resonance imaging; ARFI: Acoustic radiation force impulse; HCC: hepatocellular carcinoma; NAFLD: Non-Alcoholic Fatty Liver Disease; NASH: Non-Alcoholic Steatohepatitis; ALD: Alcoholic Liver Disease; HBV: hepatitis B virus; HCV: hepatitis C virus; HSC: hepatic stellate cells; MF: Myofibroblasts.

## Competing interests

The authors declare that they have no competing interests.

## Authors' contributions

PL carried out the systematic review and wrote a draft of the manuscript. ABar edited the manuscript, substantially expanded certain chapters, re-wrote the manuscript in its entirety after receiving the comments of the Reviewers and produced Figures. ABir collected materials for the systematic review, charted the structure of the manuscript and provided weekly supervision of the PL progress. ZY participated in the design of the systematic review, edited the final version of the manuscript and provided overall coordination. All authors read and approved the final manuscript.

## Pre-publication history

The pre-publication history for this paper can be accessed here:

http://www.biomedcentral.com/1471-230X/11/91/prepub
